# Usefulness of alien sterilizing cytoplasms for the hybrid breeding of triticale (x*Triticosecale* Wittmack): preliminary results

**DOI:** 10.1007/s13353-024-00882-z

**Published:** 2024-06-14

**Authors:** Magdalena Simlat, Tomasz Warzecha, Stefan Stojałowski, Halina Góral

**Affiliations:** 1 Department of Plant Breeding, Physiology and Seed Science, University of Agriculture in Krakow, Łobzowska 24, 31-140 Krakow, Poland; 2 Department of Plant Genetics, Breeding and Biotechnology, West Pomeranian University of Technology in Szczecin, Słowackiego 17, 71-434 Szczecin, Poland

**Keywords:** Cytoplasmic male sterility (CMS), F_1_ hybrids, Male fertility restoration, Triticale

## Abstract

To be useful for cereal breeding, cytoplasmic male sterility (CMS) should express the complete sterility of maternal lines and the full restoration of the male fertility of F_1_ hybrids. The most reliable source of sterilizing cytoplasm for triticale is *Triticum timopheevi*; however, due to the low frequency of efficient non-restorer genotypes for this cytoplasm, new sources of CMS are needed. In this study, aside from *T. timopheevi* (T) cytoplasm, three alternative CMS sources were tested: Pampa (P) from *Secale cereale* L., *Aegilops sharonensi*s (A), and *Ae. ventricosa* (V). The suitability of these cytoplasms for breeding was assessed based on the male fertility/sterility of F_1_ hybrids obtained through the manual pollination of CMS maternal lines with 36 triticale cultivars and breeding strains. About half of the hybrids with each type of cytoplasm were fully fertile and produced more than 30 grains per bagged spike. The highest percentage was found in hybrids with P cytoplasm (58.33%) and the lowest in hybrids with A cytoplasm (44.44%). Male sterility was observed in hybrids with P cytoplasm (16.67%) and A cytoplasm (16.67%) but not in hybrids with T or V cytoplasm. In terms of practical aspects, male sterility systems with P or A cytoplasm exhibit similarity in their ability to restore male fertility that differ from the T and V cytoplasms. Although all studied cytoplasms exhibited some disadvantages for breeding purposes, none should be definitively classified as unacceptable for future breeding programs regarding the development of triticale hybrid cultivars.

## Introduction

Triticale (x*Triticosecale* Wittmack) is a manmade, self-pollinated cereal crop derived by crossing wheat (*Triticum* spp.) with rye (*Secale cereale* L.). The largest producers of this species are Poland, Germany, Belarus, France, China, Canada, and the USA as well. Triticale is typically cultivated using classic (line) cultivars, although breeding research has indicated the presence of the heterosis effect in F_1_ hybrids (Oettler et al. 2001; Woś et al. 2002; Góral et al. 2005; Ferrari et al. 2018). In commercial hybrid seed production, chemical hybridizing agents (CHAs) or sterility-inducing cytoplasms can be used (Cauderon et al. 1985; Nalepa 1990; Warzecha et al. 1998; Góral 1999). However, to our knowledge, only a few hybrid cultivars have been registered in European countries (Weissmann et al. 2000; Ammar et al. 2006; Longin et al. 2012; Fossati et al. 2018). Taking into account the harmful effects of CHAs on the environment, the use of sterilizing cytoplasm seems to be the most environmentally friendly and safe for health way to conduct large-scale hybrid seed production.

Cytoplasmic male sterility (CMS) often occurs as a result of interspecies crossing, when the cytoplasm of one species is combined with the nucleus of another species. Numerous taxons of *Triticum*, *Aegilops*, and *Secale cereale* have been recognized as candidate sources of sterilizing cytoplasms for triticale. To date, the most promising source of sterilizing cytoplasm is *Triticum timopheevi*. Góral (2001) found that F_1_ hybrids obtained using *T. timopheevi* cytoplasm led to an increase in grain yield of 10–20% compared to the better parental form. However, the effectiveness of *T. timopheevi* cytoplasm is hindered by the low frequency of genotypes that maintain the male sterility of maternal lines in various environmental conditions and fully restore the fertility of F_1_ progeny (Góral et al. 2006, 2007). Meanwhile, Nalepa (1990, 2003) found that *Ae. sharonensis* cytoplasm can be used for the hybrid breeding of triticale without any significant negative effects on agricultural traits. In addition, Łapiński (2005, 2014) found that Pampa cytoplasm transferred from *S. cereale* causes male sterility in triticale. To date, the only identified source of CMS is the determinants located in mitochondrial DNA (mtDNA). These determinants have the form of the so-called chimeric genes resulting from the high recombinant activity of plant mtDNA. Most known CMS determinants contain fragments of genes encoding subunits of mitochondrial ATP synthase or are situated in close proximity to such genes. The resultant proteins encoded by these chimeric genes possess characteristics such as small size, hydrophobicity, and similarity to respiratory chain proteins; consequently, they may integrate into respiratory chain complexes, thereby disrupting mitochondrial functionality and leading to impaired pollen development (Kim and Zhang 2018).

Regardless of the source of the sterilizing cytoplasm, hybrid breeding of triticale based on the CMS system is not possible without developing a stable way to maintain the male sterility of maternal lines and obtaining pollinator lines that efficiently restore male fertility in F_1_ hybrids. In practice, at least three lines are necessary for the simplest complete CMS system: a male-sterile maternal line, a maintaining line that does not have sterility-inducing cytoplasm and is free of nuclear genes that restore fertility, and a paternal line (i.e., pollen donor) with nuclear genes that can restore the male fertility of F_1_ hybrids. Nuclear genes responsible for restoring male fertility (*Rf*) are common in higher plants, often encoding RNA-binding pentatricopeptide repeat (PPR) proteins. These proteins are imported into the mitochondria, where they prevent the accumulation of either transcripts or proteins encoded by the CMS-inducing open reading frames (ORFs) through mechanisms such as processing, editing, splicing, cleavage, or by inhibiting translation (Melonek et al. 2021). In a homozygous system, the recessive variant of the *rf* genes lacks the capacity to restore male fertility.

Unfortunately, there is no universal mechanism for maintaining sterility and restoring the male fertility of triticale with different cytoplasms. Moreover, in all sterility-inducing cytoplasms in triticale, the genetic mechanism of fertility restoration is likely based on the complex interactions of nuclear and mitochondrial genes. Obviously, each sterility-inducing cytoplasm requires an individual approach to this matter. Góral et al. (2010) suggested that, in hybrids with *T. timopheevi* cytoplasm, at least four independent nuclear genes are involved in the expression of fertility restoration. The most effective restorer genes for *T. timopheevi* cytoplasm were located on chromosome 6R, with other slightly less effective ones on chromosomes 6A and 6B. Additional restorer genes were also found on chromosomes 3A and 1B (Stojałowski et al. 2013).

Given the lack of an effective CMS system for the hybrid breeding of triticale, different sources of sterilizing cytoplasm should be tested. The present study aimed to contribute to the development of an effective CMS system by assessing a set of inbred lines with *T. timopheevi* (T), Pampa (P), *Ae. sharonensis* (A), and *Ae. ventricosa* (V) sterilizing cytoplasms. We assessed the suitability of these cytoplasms on the basis of the male fertility/sterility of F_1_ hybrids for which the CMS lines were used as a female parent.

## Materials and methods

The plant material used in this study consisted of F_1_ hybrids obtained through the manual pollination of male sterile inbreeds containing T, P, A, or V sterilizing cytoplasms with 36 cultivars and breeding strains. The pollinations were carried out in 2016–2018. The maternal lines, which were developed in the Department of Plant Breeding, Physiology, and Seed Science at the University of Agriculture in Krakow, Poland, included the following phenotypically CMS stable genotypes: BC_26_ (T), BC_9_ (P), BC_9_ (A), and BC_10_ (V) (Góral 2013). The paternal genotypes were provided by the Danko Plant Breeding Company (Choryń, Poland).

A total of 144 F_1_ hybrids were obtained and evaluated in the field conditions at the experimental station of the University of Agriculture that is located in Prusy, near Krakow. Twenty-six seeds of each F_1_ hybrid were sown manually at a distance of 20 cm in two rows, which were 2.6 m long and 40 cm apart. During the heading stage, 3–5 spikes of each plant were bagged for male fertility/sterility assessment. The appearance of anthers during flowering and the number of grains on the bagged spikes (GN/S) after harvesting were evaluated. In accordance with Góral and Jagodziński (2005) and Góral et al. (2006), both observations were expressed on a five-point scale (1—no grains/spike, 2—0.1–10.0 grains/spike, 3—10.1–20.0 grains/spike, 4—20.1–30.0 grains/spike, and 5— > 30.0 grains/spike). In addition, the correlation indexes between the analyzed traits were computed.

## Results and discussion

**Fig. 1:**
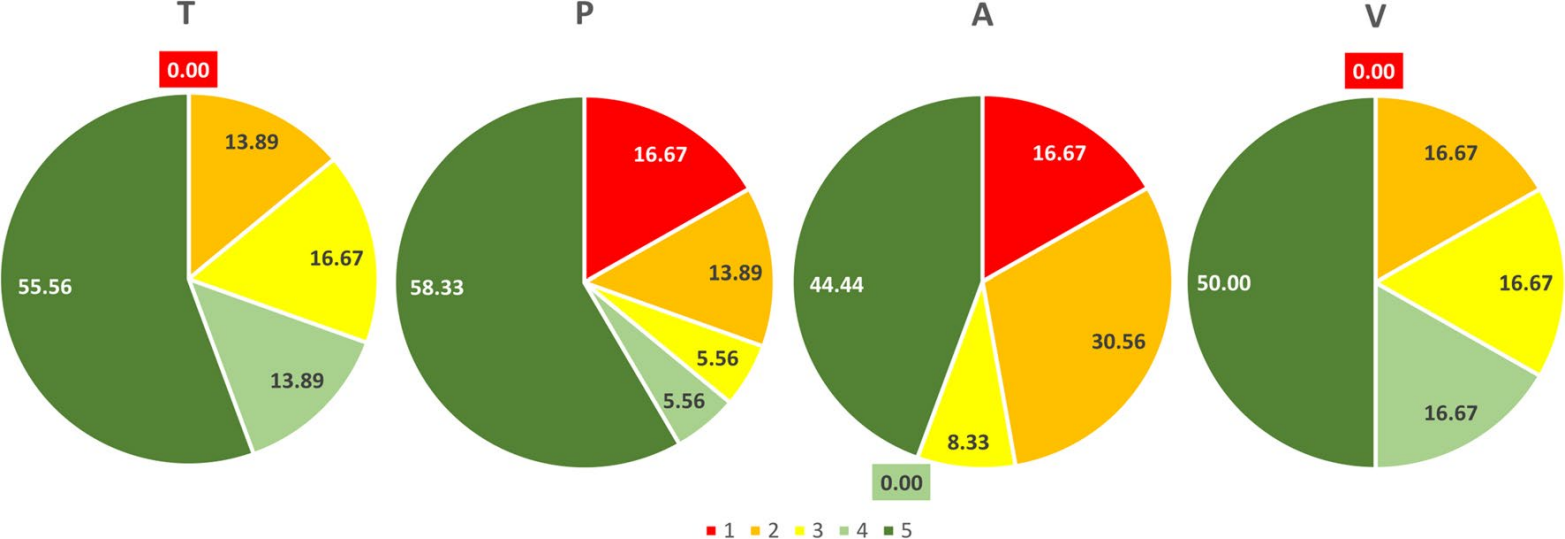
Male fertility of F_1_ hybrids with different sterilizing cytoplasms: T, *Triticum timopheevi*; P, Pampa; A, *Aegilops sharonensis*; and V, *Aegilops ventricosa*. Each chart shows the percentage share of hybrids in a given fertility class according to a five-point scale: 1—no grains/spike, 2—0.1–10.0 grains/spike, 3—10.1–20.0 grains/spike, 4—20.1–30.0 grains/spike, and 5— > 30.0 grains/spike

F_1_ hybrids with each cytoplasm differed in terms of the number of GN/S (Table 1). Male sterile hybrids, with no grains in spikes, were assessed as 1 according to the adopted scale. They were recorded for hybrids with P cytoplasm and A cytoplasm with the same frequency: 16.67%, but not in those with T cytoplasm or V cytoplasm (Fig. 1). These results indicate that male fertility/sterility depends on specific interaction between maternal and paternal genotypes, and are in line with previous studies (Góral 2013). The problem regarding the availability in European germplasm of lines that efficiently maintain male sterility in the T cytoplasm has been already reported (Góral et al. 2007; Warzecha and Salak-Warzecha 2014; Mühleisen et al. 2015). Ahirwar et al. (2013) also found a low frequency of maintainers for wheat with T cytoplasm in their study. Interestingly, in a study of Mexican breeding materials of spring triticale, Ammar et al. (2006) found a higher frequency of maintainers than restorers for this cytoplasm. Other studies also have pointed out that as in the case of T cytoplasm also in the case of the A cytoplasm there is a problem regarding the availability of genotypes maintaining male sterility (Warzecha et al. 1996, 1998; Nalepa 1990, 2003). Under the conditions of our experiment, the V cytoplasm behaved similarly to the T cytoplasm. However, no other studies have been conducted on this cytoplasm in the context of CMS.

**Table 1:** Male fertility of F_1_ hybrids with different sterilizing cytoplasm: T, *Triticum timopheevi*; P, Pampa; A, *Aegilops sharonensis*; V, *Aegilops ventricosa*

Paternal forms	CMS maternal lines							
	T		P		A		V	
	Anthers (5⁰ scale)^1^	GN/S^2^	Anthers (5⁰ scale)^1^	GN/S^2^	Anthers (5⁰ scale)^1^	GN/S^2^	Anthers (5⁰ scale)^1^	GN/S^2^
Subito	4.7	51.3	1.0	0.0	2.4	0.3	5.0	45.8
DL 593	5.0	41.6	4.6	43.5	5.0	53.2	5.0	29.9
DL 122	4.0	23.7	3.7	20.5	3.6	12.6	3.5	17.0
DL 643	5.0	45.6	5.0	67.4	2.0	2.1	4.7	27.6
DL 386	4.8	39.5	2.4	0.0	2.4	0.0	3.6	23.2
DL 1113	3.8	35.7	2.4	7.7	2.0	0.0	3.7	31.1
DL 1146	2.8	14.3	2.0	0.0	2.0	0.0	3.6	34.5
DL 402	4.8	25.8	5.0	54.4	5.0	41.4	5.0	31.9
DL 525	3.6	33.6	4.4	27.3	4.9	42.1	4.7	29.5
DL 1153	2.0	11.9	4.9	49.5	3.3	8.9	2.4	18.4
DL 1261	5.0	15.0	5.0	49.2	2.0	0.5	3.0	3.0
DL 1410	4.9	42.6	5.0	64.4	3.6	16.6	4.1	32.1
Kasyno	3.8	8.1	1.4	0.0	1.7	0.1	3.6	9.4
DC 371	4.5	16.2	1.8	0.0	1.9	0.1	3.0	13.7
DD 278	4.9	34.5	4.7	64.2	1.6	0.5	4.8	34.6
DC 719	4.4	21.7	4.8	53.9	4.8	52.5	4.8	25.9
DC 07221	3.4	9.1	1.9	0.0	1.8	0.0	2.9	7.7
DC 08065	4.0	36.7	4.1	43.1	3.9	35.8	4.6	41.4
DS 4550	4.5	44.7	4.6	53.6	4.7	54.4	4.8	50.4
DC 10047	2.8	2.8	2.4	0.1	2.2	1.7	2.6	2.6
DD 144	3.1	5.2	4.5	19.3	3.7	18.8	2.7	4.2
DD 167	4.7	43.0	1.6	1.4	3.1	8.1	4.9	46.3
DD 220	4.8	47.0	4.9	58.6	4.9	55.4	5.0	49.5
DL 1337	3.5	28.4	3.5	19.5	3.0	7.3	4.1	26.5
Porto	3.7	45.8	5.0	63.0	4.9	59.6	4.6	43.6
Orinoko	5.0	40.3	3.1	3.9	4.2	35.5	4.8	38.8
Rolando	3.3	16.0	4.8	54.0	4.3	48.5	4.1	19.4
DD 219	4.9	35.9	5.0	63.3	5.0	60.0	4.4	19.6
DD 225	5.0	34.2	4.6	64.5	4.0	35.1	4.8	31.5
DD 257	5.0	40.3	5.0	65.4	5.0	66.1	4.8	39.5
DD 608	5.0	47.2	2.8	0.5	2.7	0.0	4.8	50.4
DS 2607	1.5	1.8	3.8	45.2	4.5	33.3	1.2	1.4
DC 09176	5.0	42.3	3.9	34.4	4.4	51.5	5.0	44.0
DC 09372	3.2	15.0	4.4	45.1	5.0	52.7	4.0	17.9
DC 10252	4.8	26.9	4.7	57.3	3.0	0.0	4.9	38.3
DC 10252	5.0	42.7	4.9	53.6	3.6	0.2	5.0	37.9
Correlation coefficients								
Anthers-GN/S	0.73***		0.92***		0.92***		0.83***	

^***^Significant at the 0.001 probability level

^1^5⁰ scale for anthers classification was adopted from Góral and Jagodziński 2005

^2^Grain number per bagged spike; values are means from all plants (*n* = 7–26) of each F_1_ hybrid

The vast majority of hybrids tested in the present study produced varying amounts of grains in the bagged spikes (lowest GN/S = 0.1, highest GN/S = 67.4). Thus, although a large pool of breeding lines carried genes for restoring male fertility, the number of genes required for the efficient production of pollen grains varied depending on the cytoplasm and the pollinator. This variation may also be caused by the presence of modifier genes, differences in the penetrance or expressivity of the genes responsible for restoring pollen fertility, or a combination of these reasons (Umadevi et al. 2010). Approximately half of the hybrids produced more than 30 grains per bagged spike (score = 5). Specifically, 58.33%, 55.0%, 50.0%, and 44.44% of the hybrids with P cytoplasm, T cytoplasm, V cytoplasm, and A cytoplasm, respectively, received this score. The remaining hybrids had intermediate male fertility, producing an average of 0.1–30.0 grains per spike (scores = 2–4) (Table 1, Fig. 1). Previous studies have revealed a variable degree of fertility restoration in T cytoplasm depending on the pollen donor (Góral and Spiss 2005; Góral et al. 2006). However, Góral et al. (2007) suggested that a lack of full male fertility restoration in T cytoplasm is not a problem for the production of hybrid seeds due to the tendency of triticale to cross-pollination. Although the use of T cytoplasm in practice is not free of problems, few studies have examined the effectiveness of other sterilizing cytoplasms as sources of CMS for triticale. In addition to the CMS effect, they may also significantly expand the genetic variability of that species. Studies conducted on other species have also demonstrated the effect of the cytoplasm on the agronomic features of hybrids (Moran and Rooney 2003; Rai et al. 2008; Kibalnik and Elkonin 2020).

Although the fertility assessment in the present study was performed based on GN/S, we also conducted a visual observation of anthers during flowering, classifying the fertility of the hybrids based on their appearance (Góral and Jagodziński 2005). This type of assessment has a relatively low level of labor intensity and, as some authors have pointed out, is highly correlated with the grain number in bagged spikes (Stojałowski et al. 2004). However, in the context of triticale, to date, such observations have only been made for T cytoplasm. Our findings showed that each of these male fertility assessment methods, independent of the source of sterilizing cytoplasm, had a high correlation with the highest correlation coefficient value for hybrids with P and A cytoplasms (0.92) (Table 1). This indicates, that in the case of alternatives to T cytoplasm, a properly performed visual assessment of anthers during flowering is a reliable and valuable indicator of fertility.

In conclusion, the present study’s assessment of the male fertility restoration of F_1_ hybrids with different cytoplasms and the same paternal forms suggests that T and V cytoplasms as well as P and A cytoplasms have similar abilities in terms of restoring male fertility. A higher frequency of male fertility restoration was observed for hybrids with T and V cytoplasms compared to those with P and A cytoplasms. Furthermore, only F_1_ hybrids with P or A cytoplasm expressed full male sterility. The tested breeding materials may enable the development of CMS systems with P and A cytoplasms. For both cytoplasms, genotypes which maintain male sterility or restore male fertility were found.

## Data Availability

The data presented in this study are available on request from the corresponding author.
